# Bone Mineral Density Is a Predictor of Mortality in Female Patients with Cholangiocellular Carcinoma Undergoing Palliative Treatment

**DOI:** 10.3390/biomedicines10071660

**Published:** 2022-07-11

**Authors:** Markus S. Jördens, Linda Wittig, Christina Loberg, Lisa Heinrichs, Verena Keitel, Maximilian Schulze-Hagen, Gerald Antoch, Wolfram T. Knoefel, Georg Fluegen, Sven H. Loosen, Christoph Roderburg, Tom Luedde

**Affiliations:** 1 Department of Gastroenterology, Hepatology and Infectious Diseases, University Hospital Düsseldorf, Medical Faculty of Heinrich Heine University Düsseldorf, 40225 Düsseldorf, Germany; linda.wittig@med.uni-duesseldorf.de (L.W.); lisa.heinrichs@med.uni-duesseldorf.de (L.H.); verena.keitel@med.uni-duesseldorf.de (V.K.); sven.loosen@med.uni-duesseldorf.de (S.H.L.); christoph.roderburg@med.uni-duesseldorf.de (C.R.); tom.luedde@med.uni-duesseldorf.de (T.L.); 2 Department of Diagnostic and Interventional Radiology, University Hospital Düsseldorf, Medical Faculty of Heinrich Heine University Düsseldorf, 40225 Düsseldorf, Germany; christina.loberg@med.uni-duesseldorf.de (C.L.); antoch@med.uni-duesseldorf.de (G.A.); 3 Department of Gastroenterology, Hepatology and Infectious Diseases, Medical Faculty of Otto-von-Guericke-University, 39120 Magdeburg, Germany; georg.fluegen@med.uni-duesseldorf.de; 4 Department for Diagnostic and Interventional Radiology, University Hospital Aachen, 52074 Aachen, Germany; mschulze@ukaachen.de; 5 Department of General, Visceral and Pediatric Surgery, University Hospital Düsseldorf, Medical Faculty of Heinrich Heine University Düsseldorf, 40225 Düsseldorf, Germany; knoefel@med.uni-duesseldorf.de

**Keywords:** cholangiocellular carcinoma, CCA, osteopenia, survival, prognostic marker

## Abstract

Background: Cholangiocellular adenocarcinoma (CCA) is a rare and aggressive malignancy originating from the bile ducts. Its general prognosis is poor as therapeutic options are limited. Many patients present with advanced stages of disease, and palliative chemotherapy remains the only treatment option. Prognostic markers to assess the outcome of chemotherapeutic treatment in CCA are limited. We therefore evaluated bone mineral density (BMD) as a prognostic tool in patients with advanced CCA. Patients and Methods: We included 75 patients with advanced CCA that were treated at our academic tumor center. Prior to treatment, bone mineral density was analyzed at the first lumbar vertebra using routine CT scans in the venous phase and the local PACS (IntelliSpace PACS, Philips, Amsterdam, The Netherlands). Results: BMD was not significantly different between male and female patients but decreased with age. Patients with BMD above 167 HU have a significantly improved overall survival (474 days vs. 254 days; log-rank X^2^(1) = 6.090; *p* = 0.014). The prognostic value of BMD was confirmed using univariate (HR 2.313 (95%CI: 1.170–4.575); *p* = 0.016) and multivariate (HR 4.143 (95%CI: 1.197–14.343); *p* = 0.025) Cox regression analyses. Subgroup analysis revealed that the prognostic value of BMD was only present in female patients and not in male patients, suggesting sex-specific differences. Conclusions: Our data suggest that BMD is a valuable, easily accessible, and independent prognostic marker for overall survival in patients with advanced CCA. Furthermore, subgroup analysis showed the sex specificity of this marker, which demonstrated relevance only in female patients.

## 1. Introduction

Cholangiocellular carcinoma (CCA) is the second most common primary tumor of the liver. CCA is subdivided into those arising from the intrahepatic bile ducts, extrahepatic bile ducts, and the gallbladder [1]. Unfortunately, all cases share a poor prognosis [2]. Surgical removal represents the only curative treatment option. However, many patients display advanced or even metastasized disease stages upon initial tumor diagnosis and are only amenable to palliative treatment options. In these patients, classical cytotoxic chemotherapy represents the standard of care [1,3]. However, many patients fail to respond to this treatment. In this context, prognostic and predictive biomarkers that can differentiate patients who are likely to benefit from treatment from those patients who will not would significantly improve the clinical management of patients [4].

Alterations in bone mineral density (BMD) have been suggested to play a predictive role in various diseases. A reduction in BMD, for example, was associated with a poorer survival of these patients upon treatment of critical illnesses on intensive care units [5]. Furthermore, a correlation between BMD and survival was shown for liver diseases, for example, after liver transplantation in patients with hepatocellular carcinoma (HCC) [6]. Bone mineral density is usually determined by dual-energy X-ray absorptiometry (DXA). However, a regular DXA is not part of the routine diagnostic workup in patients with CCA. Therefore, we used existing routine CT imaging as part of tumor staging to evaluate BMD as a prognostic marker in patients receiving palliative therapy for CCA. Results from these analyses were correlated with clinical patient characteristics, such as laboratory values, to demonstrate that BMD is a predictor of mortality in patients with CCA undergoing palliative treatment.

## 2. Materials and Methods

### 2.1. Selection of Study Patients

**Figure 1:**
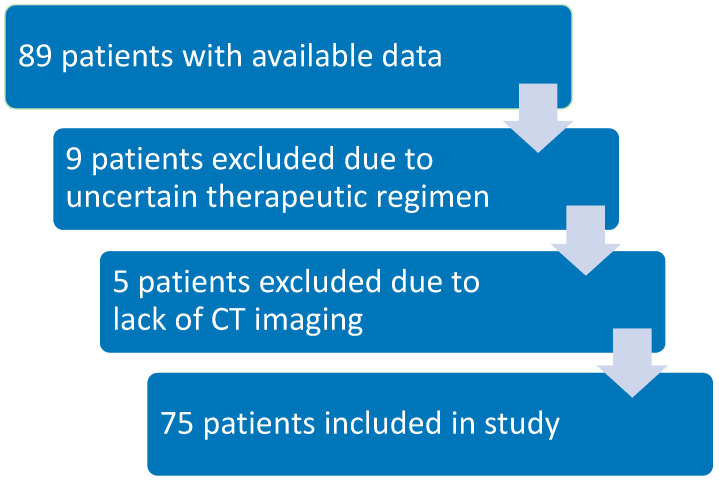
Patient selection.

In our study, we included 75 patients with advanced-stage CCA treated between 2011 and 2021 at the Department of Gastroenterology, Hepatology and Infectious Diseases or the Department for General, Visceral, and Pediatric Surgery at the University Hospital Düsseldorf [4]. In this cohort, 64 patients were treated with systemic chemotherapeutic drugs, and 11 patients received best supportive care. An interdisciplinary tumor board evaluated the individual therapeutic strategy for each patient. The CT scans used for this study were obtained during tumor staging and before the beginning of the palliative chemotherapeutic treatment or the best supportive care therapy (for detailed patient characteristics, see Table 1, for patient selection see Figure 1). The study was approved by the ethics committee of the medical faculty of the Heinrich Heine University Düsseldorf (2021-1334).

### 2.2. Patient Parameters

Patient parameters and laboratory values used for statistical analysis were obtained from the local clinical documentation system or patient charts.

### 2.3. Analysis of Bone Mineral Density

**Figure 2:**
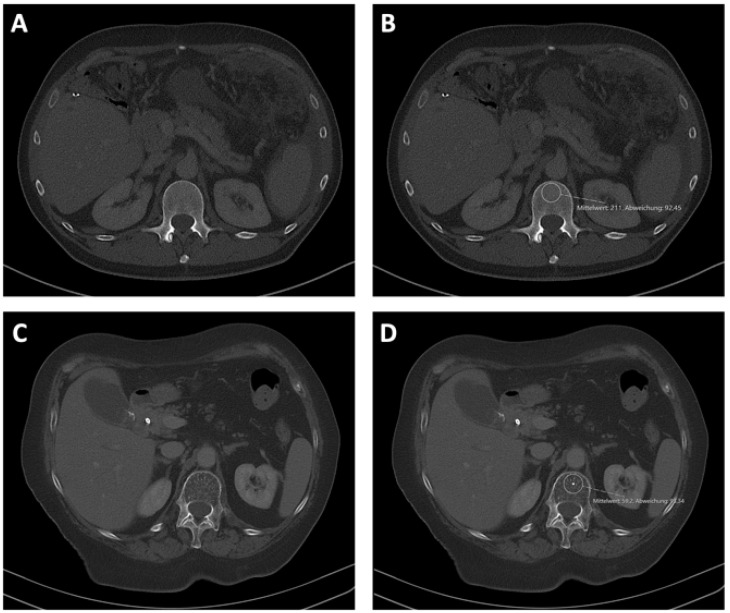
Assessment of BMD at the level of the first lumbar vertebra. BMD was assessed using CT in venous phase and the local PACS (IntelliSpace PACS, Philips, Amsterdam, The Netherlands). Examples of high (**A**,**B**) and low (**C**,**D**) BMD values with (**B**,**D**) or without (**A**,**C**) displayed ROI.

Bone mineral density (BMD) was analyzed using the local PACS (IntelliSpace PACS, Philips, Amsterdam, The Netherlands) system. BMD was measured within the first lumbar vertebra on CT scans in venous phase that had been performed during tumor staging and before the start of the palliative chemotherapy or active symptom control. BMD was measured in Hounsfield units (HU) within a manually placed region of interest (ROI) inside the trabecular bone tissue. To avoid deviations due to the venous plexus in the posterior part of the trabecular space, as well as a generally increased bone mineral density in the middle of the vertebra, BMD was evaluated in the anterior part of the trabecular bone in the upper third of L1 as previously described (Figure 2) [5]. Furthermore, the ROI was manually adjusted to avoid focal changes in bone structure, such as osteomas or bone defects.

### 2.4. Statistical Analysis

For statistical analysis, SPSS 27 (SPSS, Chicago, IL, USA) was used, as previously described in detail, unless otherwise stated [4,7]. Correlation analysis was performed using Spearman correlation, and the Shapiro–Wilk test was used to assess normal distribution. Nonparametric data were compared using the Mann–Whitney U test or the Kruskal–Wallis test. Box plots display medians, quartiles, and ranges. The impact of various patients’ characteristics on overall survival was investigated using a Kaplan–Meier curve analysis. The log-rank test was used to compare statistical differences between subgroups. As previously described, we used the optimal cut-off finder to assess the optimal cut-off values for bone mineral density [8]. To evaluate the prognostic impact of different variables regarding overall survival, we performed univariate and multivariate Cox regression analysis. The 95% confidence interval (CI 95%) and hazard ratio (HR) are displayed. *p*-values less than 0.05 are considered statistically significant.

## 3. Results

### 3.1. Baseline Characteristics of Patients

Bone mineral density was analyzed in 75 patients with cholangiocellular carcinoma treated between 2010 and 2021 with systemic chemotherapy or best supportive care at the University Hospital Düsseldorf. Out of the 75 patients included, 53.3% were male and 46.7% were female (Table 1) [4]. The median age was 70 years (range 30–87); the median BMI was 24.2 (18.5–44.3); 69% had metastatic disease; 85.3% of the patients included received palliative systemic chemotherapy (Table 1) [4]. The median BMD of all patients included was 144 HU (57.65–258 HU). The median overall survival was 224 (3–1059) days, and progression-free survival was 132 (3–916) days. Overall survival was not significantly different between male and female patients (*p* = 0.644). Progressive disease at 6 months after the start of therapy was detected in 28% of the patients.

### 3.2. Relation between Bone Mineral Density, Age, and Serum Calcium Concentrations

**Figure 3:**
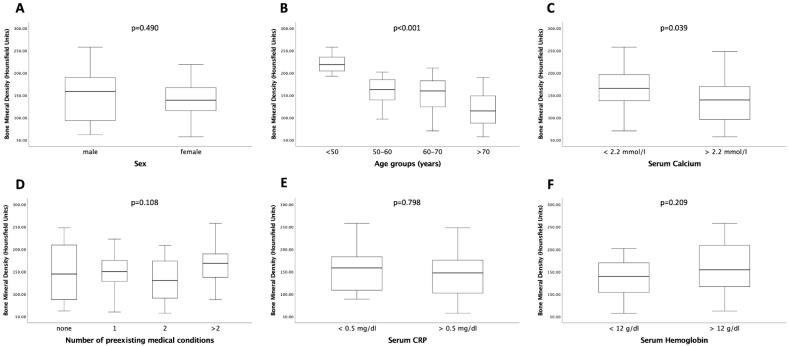
BMD is dependent on age and serum calcium. BMD was significantly different in different age groups (**B**) and serum calcium concentrations (**C**). For sex (**A**), pre-existing medical conditions (**D**), serum CRP (**E**), and serum hemoglobin (**F**), no significantly different BMD levels were observed. Box plots show medians, quartiles, and ranges.

Bone mineral density as a marker for osteopenia was measured within the trabecular space of the first lumbar vertebra (Figure 2). Patients’ sex had no influence on BMD (*p* = 0.490; Figure 3A). As expected, BMD was significantly higher in younger patients than in older patients (*p* < 0.001; Figure 3B). Surprisingly, bone mineral density was significantly lower in patients with a serum calcium concentration above 2.2 mmol/L compared to patients with a serum calcium concentration below that value (*p* = 0.039; Figure 3C). This finding might be biased by the potential calcium supplementation of patients, a factor for which information was not available in our cohort data set. BMD was independent of the number of pre-existing medical conditions (*p* = 0.108; Figure 3D). Moreover, neither patients’ serum hemoglobin (*p* = 0.209) nor serum CRP levels (*p* = 0.798) showed a significant correlation with bone mineral density (Figure 3E,F).

### 3.3. Higher Bone Mineral Density Is Associated with Improved Survival in Patients Undergoing Palliative Treatment for CCA

**Figure 4:**
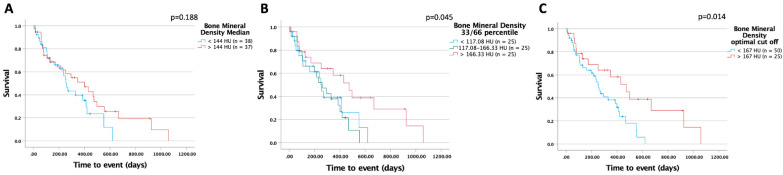
BMD is a predictor of survival in patients with advanced CCA. Patients with BMD above the median of 144 HU tend to have improved overall survival (**A**). Patients with a BMD above the 66th percentile (**B**) or above an optimal cut-off value of 167 HU (**C**) had a significantly longer overall survival.

Next, we compared the overall survival of patients with BMD above the general median of all patients (BMD > 144 HU) to patients displaying a BMD below that value. In this analysis, we identified a strong trend towards improved survival in the group of patients with higher bone mineral density (400 (95%CI: 196–604) days vs. 254 (95%CI: 213–295) days log-rank X^2^(1) = 1.736; *p* = 0.188; Figure 4A)). This trend was even more pronounced when using the 33rd and 66th percentile as cut-offs (474 (95%CI: 297–651) days vs. 260 (95%CI: 144–376) days vs. 254 (95%CI: 221–287) days; log-rank X^2^(2) = 6.190; *p* = 0.045; Figure 4B), underlining the potential prognostic value of BMD in patients receiving systemic therapy for CCA. Next, we used the *optimal cut-off finder* to identity a cut-off best distinguishing between patients with a favorable or unfavorable prognosis [8]. This analysis revealed an optimal cut-off level of 167 HU (Figure 4C); patients with a BMD above that value had significantly improved overall survival compared to all other patients (474 (95%CI: 297–651) days vs. 254 (95%CI: 211–297) days; log- rank X^2^(1) = 6.090; *p* = 0.014).

### 3.4. Bone Mineral Density of the First Lumbar Vertebra Is an Independent Prognostic Factor in Patients Undergoing Palliative Treatment for CCA

To strengthen the prognostic value of BMD at L1, we performed univariate and multivariate Cox regression analysis, including several patient characteristics (Table 2 and Table 3). Notably, the optimal cut-off of BMD of 167 HU emerged as a prognostic factor for overall survival in CCA patients undergoing palliative treatment (HR 2.313 (95%CI: 1.170–4.575); *p* = 0.016). Additionally, BMD was independent of general patient characteristics, such as age or BMI; tumor markers; markers of bone metabolism, such as serum calcium; and markers of liver function, such as bilirubin (HR 4.143 (95%CI: 1.197–14.343); *p* = 0.025) (Table 3).

### 3.5. Bone Mineral Density of the First Lumbar Vertebra Is a Sex Specific Predictor of Overall Survival in Patients Undergoing Palliative Treatment for CCA

**Figure 5:**
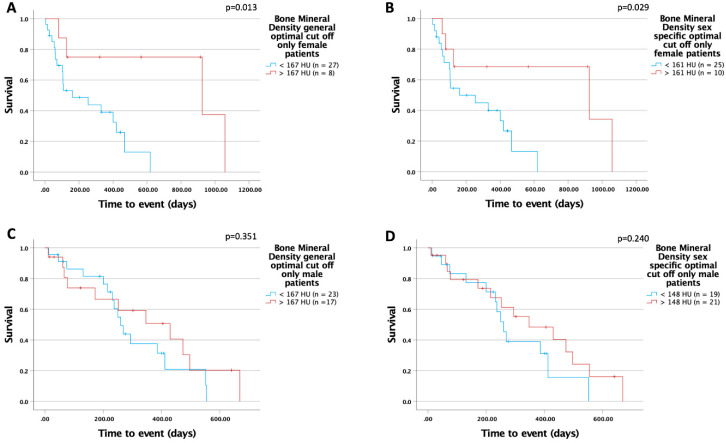
BMD is a sex-specific preoperative predictor of overall survival in patients with advanced CCA; (**A**,**C**) show the general optimal cut-off in subgroup analysis. Using a sex-specific cut-off value of 161 HU in the female subgroup, significant differences in overall survival were obtained (**B**). This was not the case in the male cohort (**D**).

Next, we performed a separate Kaplan–Meier analysis in male and female patients in our cohort. Using the optimal cut-off for the total cohort (167 HU), we identified a significantly improved overall survival in the female subgroup but not in the male subgroup (female: 925 (95%CI: 0–2078) days vs. 161 (95%CI: 0–378) days; log-rank X^2^(1) = 6.155; *p* = 0.013; male: 430 (95%CI: 164–696) days vs. 260 (95%CI: 216–304) days; log-rank X^2^(1) = 0.869; *p* = 0.351; Figure 5A,C). To further investigate potential sex differences, we used the optimal cut-off finder and determined sex-specific optimal cut-off values for BMD at L1. Interestingly, within the female subgroup, we identified a sex-specific cut-off (161 HU) with a significant benefit of higher BMD in regard to improved overall survival (female (161 HU): 925 (95%CI: 0–2085) days vs. 161 (95%CI: 0–378) days; log-rank X^2^(1) = 4.778; *p* = 0.029; male (148 HU): 347 (95%CI: 128–566) days vs. 260 (95%CI: 220–300) days; log-rank X^2^(1) = 1.383; *p* = 0.240; Figure 5B,D). Furthermore, we performed univariate Cox regression analysis separately in male and female patients. We confirmed the prognostic value of BMD in L1 for female patients but could not detect an impact in male patients (female: HR 3.761 (95%CI: 1.067–13.261); *p* = 0.039; male: HR 1.619 (95%CI: 0.720–3.641); *p* = 0.244) (Table 4).

Collectively, our data suggest a previously unknown role of BMD as a sex-specific and easily accessible prognostic marker in patients with CCA undergoing palliative treatment.

## 4. Discussion

In this study, we used existing CT scans to unravel the role of BMD as an easily accessible marker for overall outcomes in patients with CCA. By determining the BMD at the level of the first lumbar vertebra, we show that BMD is a strong and independent marker for overall survival in these patients. Subgroup analyses revealed that the prognostic value of BMD was restricted to female patients only, while, in male patients, survival was independent of the BMD.

Dual X-ray absorptiometry measurement (DXA) is the gold standard for determining BMD. It correlates with the risk of fracture and can be used in therapy monitoring for osteopenia [9]. However, DXA has no relevance in the context of staging examinations in tumor diseases. CT scans of the affected regions are performed in all tumor patients before surgery or the start of chemotherapy; therefore, they allow for the pre-therapeutic assessment of BMD in almost all patients. For the evaluation of BMD in CT scans, the Hounsfield scale is used to determine radiodensity. This determines the BMD of the patients examined without the need for additional DXA, which is associated with additional cost and radiation. The validity of routine CT scans regarding BMD has been demonstrated in recent years [10,11]. In our analyses, BMD, using an optimal cut-off of 167 HU, predicted patient survival independently of other factors such as age, BMI, or serum calcium level.

The use of BMD as a prognostic marker for OS in cancer has not been widely established but is used as a potential risk factor for the development of cancer [12,13,14]. For example, in breast cancer, a strong correlation of BMD as a marker of life-time estradiol exposure and cancer development was demonstrated in several studies. As such, high BMD was associated with increased breast cancer development [12,15], and high levels of estradiol correlated with an increased risk of the mutation of mammary cells by stimulating their mitotic activity [16]. In contrast, low levels of BMD represented a risk factor for osteoporotic diseases or cardiovascular diseases [17,18]. The exact mechanism of the effect of osteopenia on the poor prognosis of CCA remains to be elucidated. Cachexia itself, but also various cytokines such as interleucin-1, 6, and 8, which are released by tumor cells, can activate osteoclasts, which can cause osteopenia [19,20]. Furthermore, the NF-κB can also become involved through the activation of RANK/RANKL by cancer-cell-released cytokines, which is a key pathway in the progression of various digestive tract cancers [20,21,22]. Therefore, these findings suggest that osteopenia may also be associated with a poor prognosis in CCA.

As we did not detect relevant differences in overall survival between men and women, we performed subgroup analyses for BMD as a prognostic marker in advanced CCA. Strikingly, when using a sex-specific cut-off for BMD, we could exclusively identify a prognostic value of the marker in women but not in men. Our finding that sex differences are relevant for the prognostic value of BMD is interesting in light of previous findings. Recently, we identified muscle mass as a sex-specific, postoperative prognostic factor in patients receiving surgery for CCA [23]. Furthermore, Kuo et al. identified sarcopenia as a prognostic factor after liver transplantation in male patients but not in female patients [24]. In contrast, BMD seems to be a relevant prognostic factor in advanced CCA in women but not in men. These sex differences could be explained by specific hormonal changes in men and women. BMD is regarded as a marker for life-time exposure to endogenous estradiol [25]. In women, gonadal steroid levels already decline during midlife to prepubertal levels, while in men, they remain relatively stable until an advanced age [26,27]. Moreover, to have a notable impact on BMD, the levels of gonadal steroids need to substantially decrease [28]. Therefore, relevant changes in estradiol have a strong impact on bone mineral density in women [23] and might, in part, also explain the effects observed in our analyses. Testosterone, the predominant hormone in men, seems to have an especially strong effect on muscle mass [24]. Furthermore, estradiol in men is predominantly obtained through the aromatization of testosterone; therefore, levels of gonadal steroids are closely correlated [29,30]. Sex-specific changes in gonadal hormone levels are an explanation for differential changes in BMD and muscle mass in men and women. In addition, these observations might also help to explain why BMD is particularly relevant for women and, in contrast, muscle mass is more relevant in men.

Several limitations apply to our study. Due to the retrospective nature of our study, the data sets were incomplete for some patients regarding, e.g., some laboratory values and information about environmental factors such as smoking or alcohol consumption. We particularly lacked information about tumor markers (CEA, Ca19-9, AFP) in approximately 20–25% of our patients, and the precise localization of the tumor due to insufficient documentation, which could be a confounder in our analyses. Furthermore, data were not specifically obtained for our research question. Therefore, information about hormonal replacement therapy in female patients and serum levels of gonadal hormones were not documented, which might have an impact on our findings. Moreover, our data on 75 patients from a single-center would need to be confirmed in an external cohort of patients.

In summary, our study is the first to demonstrate a gender-specific role of BMD as a prognostic marker in advanced CCA. While the pathophysiologic mechanisms underlying this observation are beyond the focus of this manuscript, our data are interesting from a clinical perspective in several ways. First, we identify a subgroup of patients who may particularly benefit from targeted nutritional therapy such as vitamin D substitution. In addition, they support the need for personalized and potentially gender-specific assessments of biomarkers in the context of malignant tumors. Finally, our data support the flanking of classical chemotherapy with additional measures to maintain a healthy general and nutritional status during the initiation of therapy.

## Figures and Tables

**Table 1:** Study Cohort.

Parameter	Study Cohort
CCA patients	*n* = 75
Gender (%):	
male	53.3 (40)
female	46.7 (35)
Age (years, median, and range)	70 (30–87)
BMI class (kg/m^2^, %)	
BMI < 20	9.3 (7)
BMI 20–25	45.3 (34)
BMI 25–30	28 (21)
BMI > 30	17.3 (13)
Systemic therapy (%)	
Yes	85.3 (64)
No	14.7 (11)
Chemotherapy regimen (%)	
Gemcitabine + Cisplatin	81.3 (52)
Gemcitabine + Oxaliplatin	4.7 (3)
Carboplatin + Paclitaxel	1.6 (1)
Capecitabine Mono	1.6 (1)
Gemcitabine Mono	10.9 (7)
Tumor progression during follow-up? (%)	
Yes	28 (21)
No	72 (54)
Localization of tumor metastasis (%)	
Lymphatic	20 (15)
Vascular	8 (6)
Pulmonary	18.7 (14)
Bone	10.7 (8)
Suprarenal gland	1.3 (1)
Peritoneum	22.7 (17)
Other	16 (12)
Pre-existing medical conditions (%)	
Preceded tumor disease	24 (18)
Preceded systemic chemotherapy	1.3 (1)
Diabetes mell. Typ 2	29.3 (22)
Arterial hypertension	58.7 (44)
Hepatitis B	6.7 (5)
Hepatitis C	5.3 (4)
Alcohol abuse	1.3 (1)
Primary biliary cholangitis	2.7 (2)
Primary sclerosing cholangitis	1.3 (1)
Nonalcoholic steatohepatitis	2.7 (2)
Inflammatory bowel disease	1.3 (1)
Gastritis	24 (18)
Overall survival (days, median, and range)	224 (3–1059)
Progression-free survival (days, median, and range)	132 (3–916)
Bone mineral density (HU, median, and range)	144 (57.65–258)
AFP (mean, range)	1025.5 (1.0–31,496) ng/mL (49)
CEA (mean, range)	38.8 (0.7–653.5) ng/mL (54)
Ca19-9 (mean, range)	1584.8 (0.6–10,000) U/mL (56)

BMI: Body mass index; AFP: Alpha-fetoprotein; CEA: Carcinoembryonic antigen; CA19-9: Carbohydrate antigen 19-9; table modified after [4].

**Table 2:** Univariate Cox regression analysis for the prediction of overall survival.

Parameter	*p*-Value	Hazard Ratio (95% CI)
Gender	0.645	1.145 (0.644–2.037)
Height	0.205	0.978 (0.945–1.012)
Bodyweight	0.225	0.988 (0.969–1.008)
BMI	0.468	0.979 (0.942–1.037)
Age	0.196	1.018 (0.991–1.045)
Preceded malignancy	0.383	1.364 (0.679–2.741)
Diabetes	0.785	1.092 (0.582–2.049)
Arterial hypertension	0.381	0.774 (0.435–1.374)
Hepatitis B	0.645	0.784 (0.278–2.209)
Hepatitis C	0.085	0.402 (0.143–1.133)
Alcohol abuse	0.971	0.963 (0.132–7.051)
PBC	0.709	0.683 (0.092–5.054)
PSC	0.564	1.800 (0.244–13.299)
CIBD	0.582	0.571 (0.078–4.199)
Gastritis	0.400	1.336 (0.680–2.626)
Lymphatic metastasis	0.574	1.246 (0.578–2.688)
Vascular metastasis	0.880	1.082 (0.388–3.022)
Pulmonary metastasis	0.704	0.872 (0.430–1.767)
Osseus metastasis	0.403	1.488 (0.587–3.773)
Suprarenal gland metastasis	0.618	0.602 (0.082–4.421)
**Peritoneal metastasis**	**0.003**	**0.372 (0.194–0.713)**
**Other metastasis**	**0.006**	**0.362 (0.174–0.752)**
Sodium	0.127	0.926 (0.838–1.022)
Potassium	0.275	1.454 (0.742–2.849)
Calcium	0.230	0.287 (0.037–2.203)
Creatinine	0.649	0.979 (0.895–1.071)
**Urea**	**<0.001**	**1.024 (1.010–1.038)**
GFR	0.198	0.992 (0.979–1.004)
Uric acid	0.784	1.033 (0.818–1.305)
Bilirubin	0.318	1.066 (0.940–1.209)
ALT	0.897	1.000 (0.997–1.003)
AST	0.938	1.000 (0.996–1.004)
γGT	0.603	1.000 (0.999–1.001)
**CRP**	**0.002**	**1.124 (1.044–1.211)**
Albumin	0.207	0.646 (0.328–1.274)
**Leukocytes**	**0.003**	**1.095 (1.030–1.165)**
Hemoglobin	0.174	0.902 (0.778–1.046)
MCV	0.664	1.010 (0.967–1.054)
MCH	0.824	0.987 (0.880–1.107)
**Thrombocytes**	**0.030**	**1.003 (1.000–1.006)**
Quick	0.605	1.006 (0.983–1.029)
INR	0.841	0.918 (0.396–2.125)
aPTT	0.754	1.009 (0.955–1.066)
AFP	0.861	1.000 (1.000–1.000)
CEA	0.174	**1.001 (0.999–1.004)**
CA19-9	0.087	1.000 (1.000–1.000)
Bone mineral density	0.102	0.995 (0.989–1.001)
**Bone mineral density cut-off 167**	**0.016**	**2.313 (1.170–4.575)**

BMI: Body mass index; PBC: Primary biliary cholangitis; PSC: Primary sclerotic cholangitis; CIBD: Chronic inflammatory bowel disease; GFR: Glomerular filtration rate; ALT: Alanine aminotransferase; ALT: Aspartate aminotransferase; γGT: Gamma-glutamyltransferase; CRP: C-reactive protein; MCV: Mean corpuscular volume; MCH: Mean corpuscular hemoglobin; INR: International normalized ratio; aPTT: Activated partial thromboplastin time; AFP: Alpha-fetoprotein; CEA: Carcinoembryonic antigen; CA19-9: Carbohydrate antigen 19-9; table modified after [4].

**Table 3:** Multivariate Cox regression analysis for the prediction of overall survival.

Parameter	*p*-Value	Hazard Ratio (95% CI)
Age	0.714	0.991 (0.945–1.0939)
Body mass index	0.432	1.032 (0.954–1.115)
Calcium	0.980	0.965 (0.064–14.512)
Bilirubin	0.964	1.008 (0.706–1.439)
AFP	0.933	1.000 (1.000–1.000)
CA19-9	0.653	1.000 (1.000–1.000)
**Bone mineral density cut-off 167 HU**	**0.025**	**4.143 (1.197–14.343)**

AFP: Alpha-fetoprotein; CA19-9: Carbohydrate antigen 19-9.

**Table 4:** Univariate Cox regression analysis for the prediction of sex-specific overall survival.

Parameter	Sex	*p*-Value	Hazard Ratio (95% CI)
Height	M	0.163	0.960 (0.906–1.017)
	F	0.174	0.957 (0.898–1.020)
Bodyweight	M	0.364	0.984 (0.951–1.019)
	F	0.591	0.993 (0.966–1.020)
BMI	M	0.766	0.984 (0.883–1.096)
	F	0.850	0.993 (0.926–1.065)
Age	M	0.238	1.021 (0.986–1.056)
	F	0.351	1.020 (0.978–1.064)
Preceded malignancy	M	0.427	1.631 (0.487–5.459)
	F	0.840	1.098 (0.445–2.710)
Diabetes	M	0.497	1.325 (0.588–2.987)
	F	0.717	0.829 (0.301–2.285)
Arterial hypertension	M	0.804	0.905 (0.410–1.995)
	F	0.247	0.602 (0.255–1.422)
Hepatitis B	M	0.728	1.300 (0.297–5.702)
	F	0.747	0.717 (0.095–5.437)
Hepatitis C	M	0.220	0.463 (0.136–1.583)
	F	0.065	0.127 (0.014–1.136)
Gastritis	M	0.633	1.250 (0.500–3.123)
	F	0.324	1.734 (0.581–5.179)
Lymphatic metastasis	M	0.947	1.038 (0.345–3.119)
	F	0.690	1.250 (0.417–3.752)
Vascular metastasis	M	0.842	1.132 (0.335–3.829)
	F	0.849	1.217 (0.162–9.163)
Pulmonary metastasis	M	0.726	0.861 (0.371–2.002)
	F	0.764	1.254 (0.285–5.522)
Osseus metastasis	M	0.197	2.034 (0.692–5.983)
	F	0.664	0.638 (0.084–4.847)
Peritoneal metastasis	**M**	**0.004**	**0.211 (0.072–0.615)**
	F	0.062	0.423 (0.172–1.045)
Other metastasis	**M**	**0.004**	**0.195 (0.065–0.589)**
	F	0.203	0.512 (0.182–1.435)
Sodium	M	0.525	0.951 (0.814–1.110)
	F	0.098	0.902 (0.799–1.019)
Potassium	M	0.252	1.827 (0.651–5.127)
	F	0.999	1.000 (0.417–2.401)
Calcium	M	0.383	0.302 (0.021–4.431)
	F	0.442	0.333 (0.020–5.484)
Creatinine	M	0.056	1.687 (0.986–2.886)
	F	0.675	0.956 (0.774–1.180)
Urea	**M**	**0.005**	**1.026 (1.008–1.045)**
	F	0.056	1.041 (0.999–1.084)
GFR	M	0.263	0.990 (0.973–1.008)
	F	0.452	0.992 (0.973–1.012)
Uric acid	M	0.873	1.034 (0.688–1.554)
	F	0.811	1.041 (0.748–1.450)
Bilirubin	M	0.052	1.143 (0.999–1.309)
	F	0.801	0.967 (0.746–1.254)
AST	M	0.094	0.994 (0.988–1.001)
	F	0.260	1.004 (0.997–1.010)
ALT	M	0.146	0.995 (0.988–1.002)
	F	0.425	1.003 (0.996–1.011)
γGT	M	0.710	1.000 (0.999–1.001)
	F	0.501	1.000 (0.999–1.002)
AP	M	0.605	0.999 (0.997–1.002)
	F	0.961	1.000 (0.997–1.003)
**CRP**	**M**	**0.045**	**1.120 (1.003–1.250)**
	**F**	**0.018**	**1.140 (1.023–1,269)**
Albumin	M	0.991	1.006 (0.394–2.569)
	**F**	**0.025**	**0.269 (0.086–0.846)**
Leukocytes	M	0.192	1.092 (0.957–1.245)
	**F**	**0.003**	**1.117 (1.038–1.201)**
Hemoglobin	**M**	**0.023**	**0.767 (0.611–0.963)**
	F	0.532	0.926 (0.728–1.178)
MCV	M	0.098	1.048 (0.991–1.109)
	F	0.204	0.951 (0.881–1.027)
MCH	M	0.349	1.077 (0.922–1.258)
	F	0.080	0.857 (0.722–1.019)
Thrombocytes	M	0.141	1.003 (0.999–1.007)
	**F**	**0.047**	**1.004 (1.000–1.007)**
Quick	M	0.495	1.009 (0.984–1.033)
	F	0.299	1.032 (0.973–1.094)
INR	M	0.667	0.823 (0.340–1.995)
	F	0.710	0.351 (0.001–86.963)
aPTT	M	0.945	0.998 (0.936–1.064)
	F	0.806	0.975 (0.800–1.189)
AFP	M	0.973	1.000 (1.000–1.000)
	F	0.281	1.002 (0.999–1.005)
CEA	M	0.717	1.001 (0.997–1.004)
	F	0.167	1.002 (0.999–1.005)
CA19-9	M	0.956	1.000 (1.000–1.000)
	**F**	**0.015**	**1.000 (1.000–1.000)**
Bone mineral density	M	0.337	0.996 (0.989–1.004)
	F	0.075	0.990 (0.979–1.001)
Bone mineral density optimal cut-off 167	M	0.244	1.619 (0.720–3.641)
	**F**	**0.039**	**3.761 (1.067–13.261)**

BMI: Body mass index; PBC: Primary biliary cholangitis; PSC: Primary sclerotic cholangitis; CIBD: Chronic inflammatory bowel disease; GFR: Glomerular filtration rate; ALT: Alanine aminotransferase; ALT: Aspartate aminotransferase; γGT: Gamma-glutamyltransferase; CRP: C-reactive protein; MCV: Mean corpuscular volume; MCH: Mean corpuscular hemoglobin; INR: International normalized ratio; aPTT: Activated partial thromboplastin time; AFP: Alpha-fetoprotein; CEA: Carcinoembryonic antigen; CA19-9: Carbohydrate antigen 19-9.

## Data Availability

Data included in this analysis represent highly sensitive medical data. It is directly against German (and European) law to publish such data in a way that would allow individual patients to be identified (e.g., by providing different clinical values of one distinct patient). Data are available upon request from the Department of Gastroenterology, Hepatology and Infectious Diseases at the University Hospital Düsseldorf for researchers who meet the criteria for access to confidential data: Wissenschaft.Gastro@med.uni-duesseldorf.de.

## References

[1] Rizvi S., Khan S.A., Hallemeier C.L., Kelley R.K., Gores G.J. (2018). Cholangiocarcinoma—Evolving concepts and therapeutic strategies. Nat. Rev. Clin. Oncol..

[2] Razumilava N., Gores G.J. (2014). Cholangiocarcinoma. Lancet.

[3] Kelley R.K., Bridgewater J., Gores G.J., Zhu A.X. (2020). Systemic therapies for intrahepatic cholangiocarcinoma. J. Hepatol..

[4] Jordens M.S., Wittig L., Heinrichs L., Keitel V., Schulze-Hagen M., Antoch G., Knoefel W.T., Fluegen G., Luedde T., Loberg C. (2021). Sarcopenia and Myosteatosis as prognostic markers in patients with advanced cholangiocarcinoma undergoing palliative treatment. J. Clin. Med..

[5] Schulze-Hagen M.F., Roderburg C., Wirtz T.H., Jordens M.S., Bundgens L., Abu Jhaisha S., Hohlstein P., Brozat J.F., Bruners P., Loberg C. (2021). Decreased bone mineral density is a predictor of poor survival in critically Ill patients. J. Clin. Med..

[6] Sharma P., Parikh N.D., Yu J., Barman P., Derstine B.A., Sonnenday C.J., Wang S.C., Su G.L. (2016). Bone mineral density predicts posttransplant survival among hepatocellular carcinoma liver transplant recipients. Liver Transpl..

[7] Loosen S.H., van den Bosch V., Gorgulho J., Schulze-Hagen M., Kandler J., Jordens M.S., Tacke F., Loberg C., Antoch G., Brummendorf T. (2021). Progressive sarcopenia correlates with poor response and outcome to immune checkpoint inhibitor therapy. J. Clin. Med..

[8] Budczies J., Klauschen F., Sinn B.V., Gyorffy B., Schmitt W.D., Darb-Esfahani S., Denkert C. (2012). Cutoff Finder: A comprehensive and straightforward Web application enabling rapid biomarker cutoff optimization. PLoS ONE.

[9] Marshall D., Johnell O., Wedel H. (1996). Meta-analysis of how well measures of bone mineral density predict occurrence of osteoporotic fractures. BMJ.

[10] Schreiber J.J., Anderson P.A., Rosas H.G., Buchholz A.L., Au A.G. (2011). Hounsfield units for assessing bone mineral density and strength: A tool for osteoporosis management. J. Bone Jt. Surg. Am..

[11] Pickhardt P.J., Pooler B.D., Lauder T., del Rio A.M., Bruce R.J., Binkley N. (2013). Opportunistic screening for osteoporosis using abdominal computed tomography scans obtained for other indications. Ann. Intern. Med..

[12] Zhang Y., Kiel D.P., Kreger B.E., Cupples L.A., Ellison R.C., Dorgan J.F., Schatzkin A., Levy D., Felson D.T. (1997). Bone mass and the risk of breast cancer among postmenopausal women. N. Engl. J. Med..

[13] Nelson R.L., Turyk M., Kim J., Persky V. (2002). Bone mineral density and the subsequent risk of cancer in the NHANES I follow-up cohort. BMC Cancer.

[14] van der Klift M., de Laet C.E., Coebergh J.W., Hofman A., Pols H.A., Rotterdam S. (2003). Bone mineral density and the risk of breast cancer: The Rotterdam Study. Bone.

[15] Ganry O., Tramier B., Fardellone P., Raverdy N., Dubreuil A. (2001). High bone-mass density as a marker for breast cancer in post-menopausal women. Breast.

[16] Bernstein L., Ross R.K. (1993). Endogenous hormones and breast cancer risk. Epidemiol. Rev..

[17] Tremollieres F., Ribot C. (2010). Bone mineral density and prediction of non-osteoporotic disease. Maturitas.

[18] Tanko L.B., Christiansen C., Cox D.A., Geiger M.J., McNabb M.A., Cummings S.R. (2005). Relationship between osteoporosis and cardiovascular disease in postmenopausal women. J. Bone Miner. Res..

[19] Sakuma K., Aoi W., Yamaguchi A. (2017). Molecular mechanism of sarcopenia and cachexia: Recent research advances. Pflugers Arch..

[20] Jones D.H., Nakashima T., Sanchez O.H., Kozieradzki I., Komarova S.V., Sarosi I., Morony S., Rubin E., Sarao R., Hojilla C.V. (2006). Regulation of cancer cell migration and bone metastasis by RANKL. Nature.

[21] De Simone V., Franze E., Ronchetti G., Colantoni A., Fantini M.C., Di Fusco D., Sica G.S., Sileri P., MacDonald T.T., Pallone F. (2015). Th17-type cytokines, IL-6 and TNF-alpha synergistically activate STAT3 and NF-kB to promote colorectal cancer cell growth. Oncogene.

[22] Peng C., Ouyang Y., Lu N., Li N. (2020). The NF-kappaB signaling pathway, the microbiota, and gastrointestinal tumorigenesis: Recent advances. Front. Immunol..

[23] Montalvo R.N., Counts B.R., Carson J.A. (2018). Understanding sex differences in the regulation of cancer-induced muscle wasting. Curr. Opin. Support. Palliat. Care.

[24] Szulc P., Duboeuf F., Marchand F., Delmas P.D. (2004). Hormonal and lifestyle determinants of appendicular skeletal muscle mass in men: The MINOS study. Am. J. Clin. Nutr..

[25] Cauley J.A., Gutai J.P., Sandler R.B., LaPorte R.E., Kuller L.H., Sashin D. (1986). The relationship of endogenous estrogen to bone density and bone area in normal postmenopausal women. Am. J. Epidemiol..

[26] Finkelstein J.S., Brockwell S.E., Mehta V., Greendale G.A., Sowers M.R., Ettinger B., Lo J.C., Johnston J.M., Cauley J.A., Danielson M.E. (2008). Bone mineral density changes during the menopause transition in a multiethnic cohort of women. J. Clin. Endocrinol. Metab..

[27] Harman S.M., Metter E.J., Tobin J.D., Pearson J., Blackman M.R. (2001). Longitudinal effects of aging on serum total and free testosterone levels in healthy men. Baltimore Longitudinal Study of Aging. J. Clin. Endocrinol. Metab..

[28] Finkelstein J.S., Lee H., Leder B.Z., Burnett-Bowie S.A., Goldstein D.W., Hahn C.W., Hirsch S.C., Linker A., Perros N., Servais A.B. (2016). Gonadal steroid-dependent effects on bone turnover and bone mineral density in men. J. Clin. Investig..

[29] Longcope C., Kato T., Horton R. (1969). Conversion of blood androgens to estrogens in normal adult men and women. J. Clin. Investig..

[30] Khosla S., Melton L.J., Atkinson E.J., O’Fallon W.M., Klee G.G., Riggs B.L. (1998). Relationship of serum sex steroid levels and bone turnover markers with bone mineral density in men and women: A key role for bioavailable estrogen. J. Clin. Endocrinol. Metab..

